# The Intracellular Domain of Sortilin Interacts with Amyloid Precursor Protein and Regulates Its Lysosomal and Lipid Raft Trafficking

**DOI:** 10.1371/journal.pone.0063049

**Published:** 2013-05-21

**Authors:** Miao Yang, Balaji Virassamy, Swarna Lekha Vijayaraj, Yoon Lim, Khalil Saadipour, Yan-Jiang Wang, Yan-Chuang Han, Jin-Hua Zhong, Carlos R. Morales, Xin-Fu Zhou

**Affiliations:** 1 School of Pharmacy and Medical Sciences, Sansom Institute, University of South Australia, Adelaide, Australia; 2 Department of Human Physiology, Flinders University of South Australia, Adelaide, Australia; 3 Department of Neurology, Daping Hospital, Third Military Medical University, Chongqing, China; 4 Department of Anatomy and Cell Biology, McGill University, Montreal, Quebec, Canada; University of Memphis, United States of America

## Abstract

The processing of Amyloid precursor protein (APP) is multifaceted, comprising of protein transport, internalization and sequential proteolysis. However, the exact mechanism of APP intracellular trafficking and distribution remains unclear. To determine the interaction between sortilin and APP and the effect of sortilin on APP trafficking and processing, we studied the binding site and its function by mapping experiments, colocalization, coimmunoprecipitation and sucrose gradient fractionation. We identified for the first time that sortilin interacts with APP at both N- and C-terminal regions. The sortilin-FLVHRY (residues 787–792) and APP-NPTYKFFE (residues 759–766) motifs are crucial for the C-terminal interaction. We also found that lack of the FLVHRY motif reduces APP lysosomal targeting and increases APP distribution in lipid rafts in co-transfected HEK293 cells. These results are consistent with our *in vivo* data where sortilin knockout mice showed a decrease of APP lysosomal distribution and an increase of APP in lipid rafts. We further confirmed that overexpression of sortilin-FLVHRY mutants failed to rescue the lysosomal degradation of APP. Thus, our data suggests that sortilin is implicated in APP lysosomal and lipid raft targeting via its carboxyl-terminal F/YXXXXF/Y motif. Our study provides new molecular insights into APP trafficking and processing.

## Introduction

Amyloid precursor protein (APP) contributes to the production of beta-amyloid (Aβ), which is a major component of senile plaques in the brain of Alzheimer’s disease (AD) patients [1], [2]. The processing of APP to Aβ involves numerous steps, including APP sorting, transport, internalization and sequential proteolysis [3], [4]. Newly synthesized APP in endoplasmic reticulum (ER) is sorted through the trans-Golgi-network (TGN), trafficked to the cell surface membrane [5], and internalized via its NPTY motif near the C terminus of APP into endosome/TGN for recycling or into lysosome for degradation [6], [7]. Aβ is generated by β- and γ-secretase sequential cleavage of APP in subcellular compartments [8]. Altered routing of APP trafficking and distribution in neurons might lead to the amyloidogenic pathway, which is implicated in the pathology of AD. Hence, the intracellular distribution and transport of APP are critical for Aβ production. Although several APP binding factors have been reported, for example, Huntingtin and kinesin light chain are involved in APP axonal transport in neurons [9], [10], [11], the exact mechanism of APP intracellular trafficking and distribution remains unclear.

Sortilin is important in neuronal functions [12] and shares genetic similarity with other Vps10p family members, such as SorLA, SORCS1 and SORCS2 [13]. Its extracellular domain (ECD) contains a homologous sequence to yeast vacuolar protein sorting 10 protein (Vps10p) [14]. The intracellular domain (ICD) is involved in protein internalization and sorting [12], [13]. Three motifs, F/YXXXXF/Y, YSVL and HDDSDEDLLE (dileucine motif), have been identified in ICD [15]. They might mediate protein sorting, trafficking and internalization through binding to adaptor proteins, such as AP-1, AP-2 and GGA [16]. Since not all of these motifs are present in SorLA, SORCS1 and SORCS2 [17], [18], it raises the possibility that sortilin may have diverse functions in cellular events. This is also backed by evidence that SorLA is down-regulated [19] but sortilin is up-regulated in AD [20]. SorLA is reported to retain APP in Golgi, this can lead to decreasing Aβ production. Given that SorLA and sortilin are members of the same family, the question is if up-regulation of sortilin might act as a compensation mechanism for the down-regulation of SorLA in AD. Moreover, lack of sortilin-ICD prevents sorting of acid sphingomyelinase (ASM) to lysosomes [21]. In addition, sortilin interacts with HAP1, a protein facilitating axonal trafficking of the precursor of neurotrophin [22]. Taken together, these studies suggest that sortilin may be involved in APP trafficking and processing. Hence, it is important to further determine how sortilin affects APP trafficking and processing and to understand the underlying molecular mechanisms.

## Materials and Methods

### Animal

All procedures involving animals were approved by the Animal Welfare Committee of Flinders University (Ethics No. 703/09) and the Animal Ethics Committee of SA pathology (Ethics No. 15b/12), and were undertaken according to the guidelines of the National Health and Medical Research Council of Australia. The use of genetically modified animals was approved by the Biosafety Committee of Flinders University and University of South Australia. All animals were kept under standardized barrier breeding conditions with free access to water and food. PCR was used for genotyping of sortilin knockout mice with 5′-TCAGGAATGGCATTCTCAG-3′/5′-AGTGCTGTCTCCAACCCAGGAC-3′ for wild type and 5′- CTCAGGAATGGCATTCTCAG-3′/5′- AAGTCGTGCTGCTTCATGTG-3′ primers for knockout animals.

### Cell Culture

Human embryonic kidney 293 cells (HEK293) were obtained from the American Type Culture Collection (ATCC; Rockville, MD) and maintained in DMEM medium (Invitrogen) supplemented with 10% fetal bovine serum (FBS) and 2 mM glutamine. Mouse cortical neurons were prepared and cultured as described [22].

### Plasmid Constructs

The constructs of sortilin–CFP were made by PCR, amplifying Xho1 or EcoR1-BamH1 fragments with the primers 5′-GTCTCGAGCCACCATGGCGCCGGGCGAGGACGAGG-3′/5′- TTGGATCCTCCARGAGGTCCTCRTCTG-3′ or 5′- GTCTCGAGCCTTCCCCCACAGACATATTTC-3′ or 5′- GTCTCGAGGACAATGCCTCGATCATCTGAG-3′ for Sort 78–831, Sort 78–786 and Sort78–385; 5′- GTCTCGAGCCACCATGGTCTATTCCAAGTCTTTGGAC-3′/5′- GTCTCGAGCCTTCCCCCACAGACATATTTC-3′ for Sort385–786; 5′-TTGAATTCGCCACCATGGTTCCAATTATCCTGGCCATC-3′/5′-TTGGATCCTCCARGAGGTCCTCRTCTG-3′ for Sort756–831; 5′-TCGAATTCGCCACCATGAAGAAATATGTCTGTGGGGGA-3′/5′-TTGGATCCTCCARGAGGTCCTCRTCTG-3′ for Sort779–831 from human sortilin pcDNA3.1-myc/His and cloning into pECFP-N1 (Clontech). To construct sort del.MS2, sort del.MS1, sort del-MS1/MS2 and sort MS1/MS2 CFP plasmids, PCR and overlapping PCR were performed, amplifying the EcoR1-BamH1 or EcoR1 fragments with the primers 5′- TTGAATTCGCCACCATGGTTCCAATTATCCTGGCCATC-3′/5′- CTGTGGATCCTGATAACCACTTTTATTAGTGTG-3′ for Sort del.MS2; 5′- TTGAATTCGCCACCATGGTTCCAATTATCCTGGCCATC-3′/5′-ACACCATTGGCCTCTGCATGCTGCACAATGAGCACTCCTGCT-5′, 5.- CAGCATGCAGAGGCCAATG-3′/5′- TTGGATCCTCCARGAGGTCCTCRTCTG-3′ for Sort del.MS1; 5′- TTGAATTCGCCACCATGGTTCCAATTATCCTGGCCATC -3′/5′- ACACCATTGGCCTCTGCATGCTGCACAATGAGCACTCCTGCT-3′, 5′- CAGCATGCAGAGGCCAATG-3′/5′- CTGTGGATCCTGATAACCACTTTTATTAGTGTG-3′ for Sort del.MS1/MS2; 5′- TTGAATTCGCCACCATGGTTCCAATTATCCTGGCCATC-3′/5′- TGGGAATTCGTTCCAAGAGGTCCTCATCTGAGTCATCTGTGTCCAAAGCATCCACAC-3′ for Sort MS1/MS2, and cloning into pECFP-N1. The constructs of APP-YFP were derived by cloning PCR products generated from human APP770-YFP pcDNA3.1 with the primers 5′- GCGCCTCGAGGCCACCATGCTGCCCGGTTTGGCACTG-3′/5′- GCGCGAATTCG ATTCATGCGCTCATAAATCAC-3′ or 5′- GCGCGAATTCG AACCACCTCTTCCACAGACTC -3′ or 5′- GCGCGAATTCGAACATCCATCCTCTCCTGGTG-3′ for APP 1-542, APP 1-287 and APP 1-141; 5′- GCGCCTCGAGGCCACCATGGATGTTTGCGAAACTCATC-3′/5′- GCGCGAATTCGAACCACCTCTTCCACAGACTC-3′ for APP 141-287; 5′- GCGCCTCGAGGCCACCATGAATCAGTCTCTCTCCCTG -3′/5′- TGGGAATTCG CATCTTCACTTCAGAGATCTC-3′ for APP 547-671; and 5′- TGGCTCGAGGCCACCATGACAGTGATCGTCATCACCTTG-3′/5′- TGGGAATTCGGTTCTGCATCTGCTCAAAG-3′ for APP 713-770, in the Xho1 and EcoR1 sites of pEYFP-N1. The integrity of all constructs was determined by sequencing. APP770-YFP pcDNA3.1 and APP695 pcDNA3.1 were gifts from Prof. Goldstein (UCSD) and Dr. Ma (Soochow University), respectively. P75CFP-YFP pcDNA3.1 was a gift from Prof. Coulson (The University of Queensland, Australia). Sortilin pcDNA3.1-myc/His (full-length) and Sortilin-T (deleting intracellular domain) pcDNA3.1-myc/His plasmids were gifts from Dr. Morales (McGill University).

### Immunocytochemistry (ICC) and Colocalization

Antibodies: mouse monoclonal APP antibody (22c11) was purchased from Millipore. EEA1 (early endosome marker), mannose 6 phosphate receptor (late endosome marker), Giantin (Golgi complex marker), and Lamp1 (lysosome marker) antibodies were purchased from Abcam (Cambridge, USA). Sortilin antibody (rabbit, OSC-203) was purchased from Osenses (Australia). Mouse monoclonal Aβ antibodies (6E10 and 4G8), Flotillin-1 (lipid raft marker) antibody and all fluorescent conjugated secondary antibodies were purchased from Sigma. Cortical neurons prepared from neonates were used for ICC as described [22]. For colocalization analysis, Leica SP5 confocal microscopy was used with a 63/1.4 NA oil immersion lens. Images of fluorochromes (Cy3/Alexa488/DAPI or YFP/Cy5/CFP) were obtained sequentially using Leica SP5 colocalization model. Quantitative assessment of colocalization between Cy3 and Alexa 488 or YFP and Cy5 fluorescence signals was performed by calculating the overlap coefficient (ranging from 0%, minimum colocalization, to 100%, maximum colocalization), using the Leica Application Suite software. An average of 20 cells was analysed in each experiment (three preparations/experiment), and the overlap coefficient (%) was calculated.

### Transfection

Transfection of HEK293 cells was performed as described [22]. Transfection with YFP or CFP constructs served to monitor transfection efficiency. The percentage of fluorescent cells was calculated using IX71 microscopy and subjected to normalization.

### FRET Acceptor Bleaching (FRET)

FRET was performed as described [22]. The p75CFP-YFP was used as positive control. A pair of proteins such as APP-YFP and pECFP (or BDNF-CFP) which show no interaction was used as a negative control. Each value of *FRET _eff_* from the tested samples including positive, negative and background controls were determined according to a mean of six individual tests from different viewing fields. The results were summarized from three different experiments. To evaluate the FRET data, the *FRET_eff_* result from the negative control was used as a cut-off in this study.

### Coimmunoprecipitation (co-IP) and Western Blot (WB)

Co-IP and WB were performed as described [22]. Briefly, Co-transfected HEK293 cells were prepared in radio immunoprecipitation assay (RIPA) buffer containing 2 mM PMSF and protease inhibitors (Roche) for cell lysates. The protein concentration of the lysates was determined using BCA protein assay Kit (Thermo Scientific, Rockford, USA). Lysates (250 µg) were precleared by incubation with protein G bead at 4°C for 1 hour and then incubated with protein G bead-immobilized antibodies at 4°C overnight. The beads were washed five times and boiled in loading buffer for WB. Protein G beads bound with rabbit or mouse IgG and CFP or YFP were used as negative controls. For WB, lysate proteins (50 µg) were analysed by 10% SDS-PAGE and transferred to nitrocellulose membrane (GE). Corresponding primary antibodies (1∶1000) were incubated with blots at 4°C overnight. Horseradish peroxidase-conjugated (HRP) secondary antibodies (1∶2000) were used for detection. β-Actin was used as a loading control. Imaging was performed using ECL (GE). ImageJ (NIH) was used for quantitative analysis.

### Lipid Raft Fractionation

Lipid rafts were prepared from transfected HEK293 cells in two 10 cm culture dishes and mouse brains as described previously [23], [24] with some modifications. Briefly, transfected cells or mouse brain were resuspended in 0.5 ml of lysis buffer containing 50 mM tris-HCl, pH 7.4, 150 mM NaCl, 5 mM EDTA, 1 mM PMSF, and 0.5% Triton X-100 supplemented with Complete protease inhibitor cocktail (Roche). Cells or brain tissue were homogenized by passages through a 25-gauge needle seven times. The concentration of total protein was measured by the Bio-Rad protein assay kit II (Bio-Rad). A total of 4 mg of protein from the homogenates was mixed with lysis buffer containing sucrose to yield a final concentration of 45% sucrose in 1 ml and laid at the bottom of an ultracentrifuge tube. To form a discontinuous sucrose gradient, Lysis buffer containing 35% (2 ml) and 5% (2 ml) sucrose were sequentially laid over the homogenates. The samples were ultracentrifuged at 44,000 rpm in a Beckman SW55 rotor at 4°C for 17 hours. Ten 0.5 ml fractions were collected from top to bottom of the gradient sample. Equal volumes (15 µl) of each fraction were subjected to SDS-PAGE for WB.

### Statistical Analysis

All statistical analyses were performed with SPSS software (version 19.0). Data were expressed as means ± SEM and *p*<0.05 was considered significant. Variables between groups were determined by independent t-test or ANOVA.

## Results

### Sortilin is Associated with APP in vitro and in vivo

**Figure 1:**
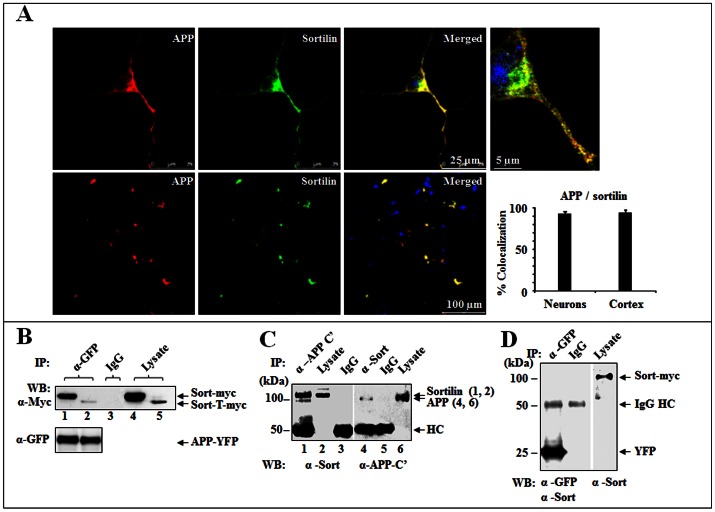
Sortilin and APP are allied *in vitro and in vivo*. (**A**) Colocalization of sortilin and APP. Mouse cortical neuron (upper) and brain cortex (lower) were immunostained for APP (red) and sortilin (green). Colocalization of sortilin and APP was indicated in merged panels (yellow). DAPI stained cell nuclei (blue). Plotted colocalization: 92% mean± SEM, n = 20, in cortical neurons and 95% mean± SEM, n = 3, in brain cortexes. Scale bar 25 µm for cortical neurons, 5 µm for enlarged image and 100 µm for brain cortex. (**B**) Co-IP of sortilin with APP in co-transfected HEK293 cells. HEK293 cells growing in 10 cm culture dishes were co-transfected with APP770-YFP/Sort-FL-myc/His (lane 1, 4) or Sort-T-myc/His (lane 2, 5). Cell lysates were immunoprecipitated with rabbit anti-GFP (α-GFP) for APP and blotted with mouse anti-Myc (α-Myc) for sortilin. Mixed lysates were used for IgG (lane 3). Sort-FL-myc.His (Sort-myc), Sort-T-myc.His (sort-T-myc) and APP770-YFP (APP-YFP) are indicated by arrows. (**C**) Co-IP of sortilin with APP in APPSwe/PS1dE9 transgenic mouse brain lysate. Mouse brain lysates were subjected to immunoprecipitation with rabbit anti-APP C’ (α-APP C’) and blotted with rabbit anti-sortilin (α-Sort) (left panel) or immunoprecipitation with α-Sort and blotted with α-APP C’ (right panel). Sortilin and APP are indicated by arrows. (**D**) Control for Co-IP using pEYFP. HEK293 cells were co-transfected with pEYFP/Sort-myc. Co-IP was performed using α-GFP and blotted with α-sort and α-GFP. Rabbit IgG (IgG) was used as a control for non-specific binding.

To investigate if sortilin is involved in APP processing, we first determined whether endogenous APP colocalized with sortilin in primary cortical neurons. Double-labelling of cortical neurons and frozen cerebral cortex sections showed colocalized APP and sortilin as punctual distribution in perinuclear region and axons. Approximately 92% and 95% of APP was highly colocalized with sortilin in either cortical neurons or cerebral cortex, respectively (Fig. 1A), suggesting an interaction between sortilin and APP. To further test this hypothesis, we performed co-IP using HEK293 cells co-expressing APP/full-length sortilin-myc/His (Sort-FL) or APP/sortilin-T-myc/His (Sort-T, a truncated form lacking the intracellular domain). Our results showed that both Sort-FL and Sort-T were immunoprecipitated by APP770-YFP, with rabbit anti-GFP antibody but did not show in negative controls immunoprecipitated with either rabbit IgG (Fig. 1B) or pEYFP (Fig. 1D). Sortilin and APP from brain homogenates of APPSwe/PS1dE9 transgenic mice (Jackson Laboratory) were also detected in the APP or sortilin immunoprecipitated complexes, but not found in the negative control (rabbit IgG) (Fig. 1C). Therefore, the interaction between sortilin and APP is present either *in vitro* or *in vivo*, suggesting that the interaction may have significant influence on cellular sortilin/APP events.

### Determining Sortilin and APP Binding Sites

**Figure 2:**
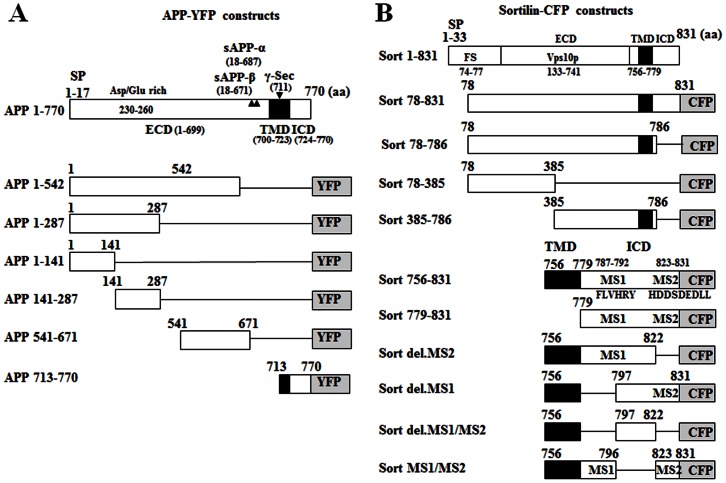
A schematic diagram of APP-YFP and sortilin-CFP constructs. The region of 78–831 aa is chosen for cloning based on that mature sortilin receptor, a bioactive form is released by furin cleavage from its proform at amino acid (aa) residue 77 in neurons (see results). The region cloned into pEYFP-N1 or pECFP-N1 is indicated by open and solid boxes. The line indicates the deleted region. Abbreviation: signal peptides: SP; extracellular domain: ECD; transmembrane domain: TMD; intracellular domain: ICD; amino acid: aa; gamma secretase: γ-Sec; furin cleavage site: FS; Motif 1: MS1 (^787^FLVHRY^792^); Motif 2: MS2 (^823^HDDSDEDLL^831^); deletion: del.; Arrowhead: α or β secretase cleavage site.

To further characterize the interaction between sortilin and APP, we constructed sortilin and APP clones harbouring varied regions in pECFP-N1 and pEYFP-N1 vectors for FRET, co-IP and cellular studies according to taxonomic and functional structures. Since mature sortilin receptor, a bioactive form, is released by furin cleavage from its proform at amino acid (aa) residue 77 in neurons [15], we selected the mature region (78–831 aa) for cloning. Due to lacking the signal peptide and/or transmembrane domain (TMD) in some constructs, they might express a misfolded form to some extent, which could interfere with protein-protein interaction and protein function. To determine if constructs contain a misfolding, we performed the N-terminal fusion to CFP or YFP so that protein misfolding would result in a loss of fluorescence [25]. We found all APP and sortilin constructs showed a bright fluorescence in transfected HEK293 cells, and sortilin constructs such as Sort 78–831 and Sort 756–831 represented an intracellular distribution similar to Sortilin-FL (data not shown), suggesting that these constructs rarely have a protein misfolding and incorrect sorting. Thus, these constructs were used for this study. The schematic diagrams depicting the constructs were shown in Figure 2A and 2B.

**Figure 3:**
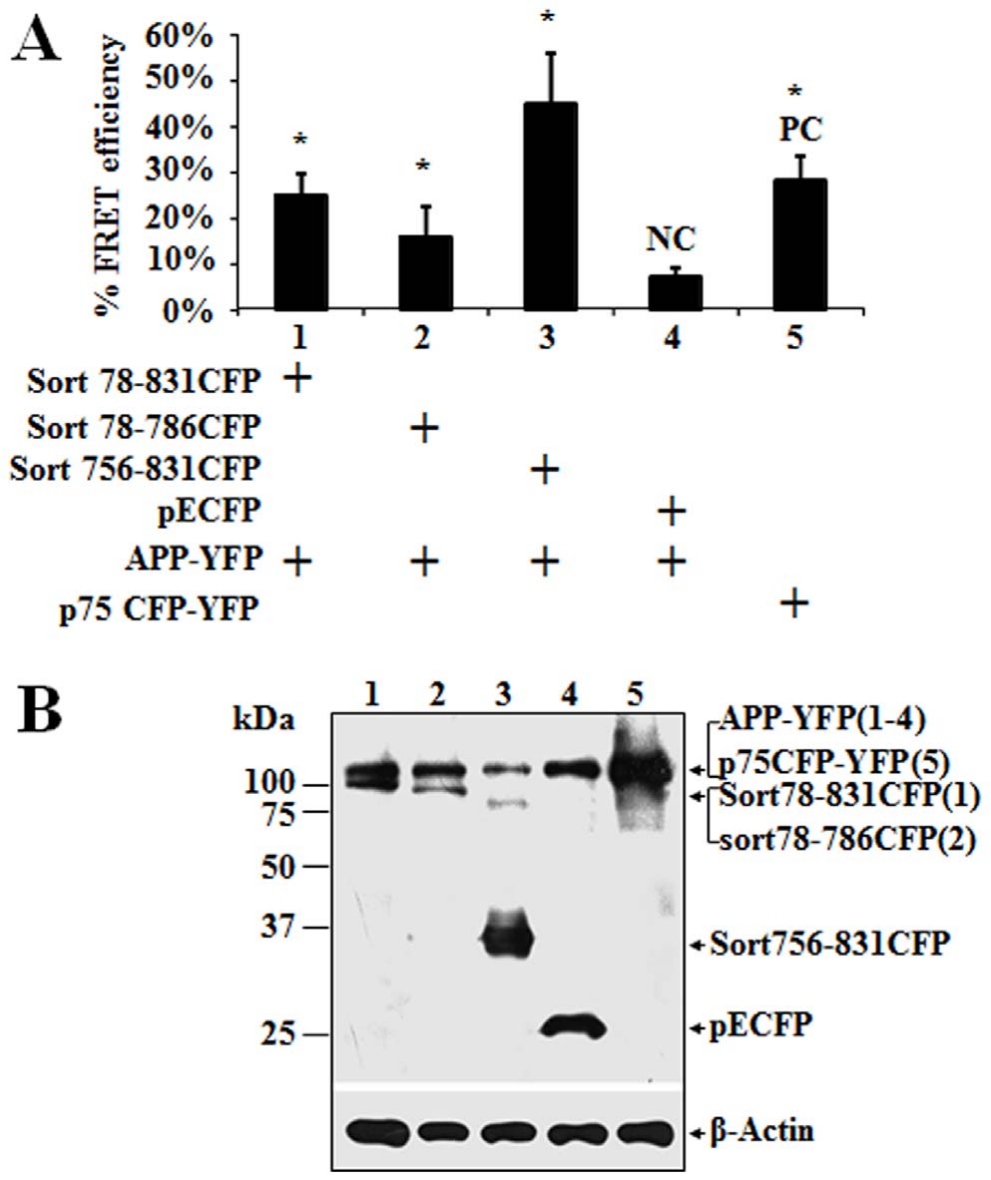
FRET analysis of the interaction between sortilin and APP. HEK293 cells co-transfected with APP770-YFP (APP-YFP) and different sortilin-CFP constructs were used for FRET. (**A**) FRET efficiency representing the protein-protein interaction was determined from a photobleached region of interest (ROI). (**B**) Expression levels for constructs used in FRET were determined by Western blot with gt-α-GFP. β-Actin was blotted with mouse-α-actin for sample equal loading. NC: negative control. PC: positive control. Three independent experiments were performed. Bars represent mean±SEM (n = 6 ROI×3). The star (*) indicates *p*<0.01.

To identify the binding sites between sortilin and APP, we performed a FRET using HEK293 cells coexpressing APP770-YFP and sortilin-CFP proteins. Three sortilin-CFP DNA constructs, named Sort 78–831 (containing the mature region of sortilin), Sort 78–786 (by removing the entire intracellular domain (ICD) except the first N-terminal 7 amino acids of the ICD) and Sort 756–831(comprising the transmembrane domain (TM) and ICD) were co-transfected with APP770-YFP into HEK293 cells, respectively (Fig. 3). Although all three sortilin CFP constructs together with APP770-YFP were expressed in HEK293 cells in a similar manner, the significant FRET efficiency (*FRET_eff_*) generated by these constructs from high to low is Sort 756–831(40% *FRET_eff_*), Sort 78–831(25% *FRET_eff_*) and Sort 78–786 (15% *FRET_eff_*), compared with negative control (NC). As Sort-T showed an interaction with APP (Fig. 1B), the less efficient binding of the N-terminus (78–786 aa) of sortilin to APP is unlikely due to the first seven amino acids of ICD overlapping between Sort 78–786 and Sort 756–831 and/or an alteration of the C-terminal binding site. Thus, this data suggests that both N- and C-termini of sortilin may interact with APP, but that the C-terminus has much stronger interaction than the N-terminus.

**Figure 4:**
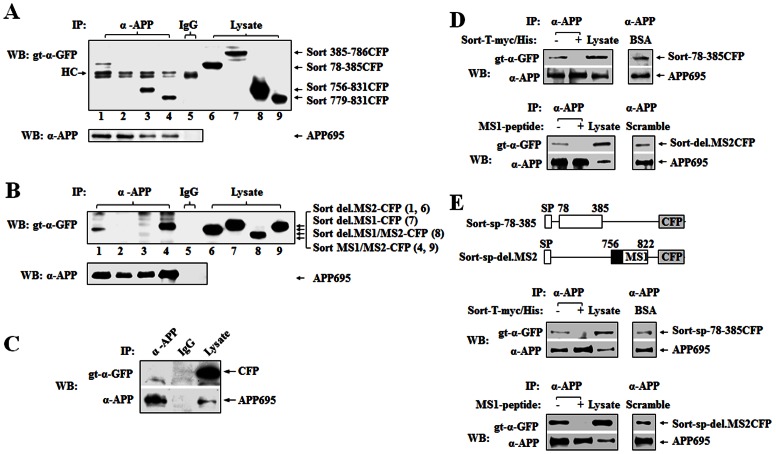
Mapping sortilin binding to APP. (**A**) Identifying sortilin binding sites to APP. HEK293 cells were co-transfected with APP695-pcDNA3.1 (APP695) and Sort 78–385-CFP (lane 1) or Sort 385–786-CFP (lane 2), or Sort 756–831 (lane 3), or Sort 779–831-CFP (lane 4). Cell lysates were immunoprecipitated with mouse anti-APP, 22c11, (α-APP, lanes 1–4) and mouse IgG (mixed lysates, lane 5), and blotted with goat-anti-GFP (gt-α-GFP). Lysates were used as input (lanes 6–9). Sort 78–385CFP (61 kDa), Sort 385–786CFP (71 kDa), Sort 756–831CFP (35 kDa) Sort 779–831-CFP (32 kDa), APP695 (100 kDa) and IgG heavy chain (HC, 50 kDa) are indicated by arrows. (**B**) Determining sortilin C-terminal FLVHR motif (MS1) binding to APP. APP695 was used with Sort del.MS2-CFP (lanes 1, 6) or Sort del.MS1-CFP (lanes 2, 7) or Sort del.MS1/MS2-CFP (lanes 3, 8) or Sort MS1/MS2 (lanes 4, 9) for co-transfection. Co-IP was performed as (A). Sort del.MS2-CFP (34 kDa), Sort del.MS1-CFP (34 kDa), Sort-del.MS1/MS2-CFP (32 kDa), Sort MS1/MS2-CFP (33 kDa) and APP695 in precipitated samples and lysate are indicated by arrows. (**C**) Control for Co-IP using pECFP. HEK293 cells were co-transfected with pECFP/APP695. Co-IP was performed using α-APP and mouse IgG (IgG) as indicated. The blot was probed with α-GFP (upper) and re-probed with α-APP (lower). Lysate was used as input. APP695 and CFP (27 kDa) are indicated by arrows. Mouse IgG was used as a control for non-specific binding in all co-IP. (**D and E**) Sort-T-myc/His and MS1 peptide (CGGRFLVHRYSVLQQ; Peptide-2.0, Chantilly, VA) corresponding to the amino acids 783–797 of sortilin (GenBank Accession No. NM_002959) prevent sortilin N or C terminal constructs binding to APP in the competition binding assay, respectively. Sort-78–385CFP and Sort-del.MS2CFP (**D**) and two new sortilin constructs (termed Sort-sp-78–385-CFP and Sort-sp-del.MS2CFP) created by overlapping PCR and cloned in pECFP (**E**) were used in the competition binding assays. The integrity of the constructs was confirmed by DNA sequencing. To purify Sort-T-myc/His, HEK293 cells growing in 3×10 cm culture dishes were transfected with Sort-T-myc/His for 48 h. Cells were harvested in cold PBS containing 1 mM PMSF and protease inhibitor (Roche), sonicated on ice and centrifuged at 15000 rpm at 4°C for 10 minutes. The supernatant was loaded into Ni-NTA column (Qiagen) and eluted in elution buffer after washing. The elute protein was concentrated by Amicon Ultra- Centrifugal Filters (Millipore) and the protein was recovered in 50 µl of PBS pH 8, containing 1 mM PMSF and protease inhibitor. For competition binding assays, HEK293 cells (1×10 cm dish) were transfected with APP695 or each sortilin construct for 48 hours and harvested for preparing cell lysates. The lysate containing APP695 was incubated with 50 µl (+) of the purified Sort-T-myc/His protein or with 10 µM (+) of MS1 peptide at 4°C for 1 hour and then combined with the lysate containing Sort-78-385CFP or Sort-del.MS2CFP or Sort-sp-78-385-CFP or Sort-sp-del.MS2-CFP, and the incubation was continued for 2 hours at 4°C with rotation. The lysates were then immunoprecipitated with α-APP and blotted for Sort-78-385 and Sort-del.MS2 by gt-α-GFP, respectively. Cell lysate was used as input. Scramble MS1 peptide (Scramble, 10 µM) and BSA (10 µM) were used as control for non-specific competition. Sortilin constructs and APP695 from co-IP samples and inputs are indicated by arrows.

Given the results that both sortilin-ICD and ECD interact with APP, we subsequenty narrowed down the sortilin binding sites to APP by co-IP. We found that Sort 78–385, Sort 756–831, and Sort 779–831 were present in the samples immunoprecipitated with APP antibody (22c11) but not Sort 385–786 which contains the C’-terminal portion of the Vps10p domain (Fig. 4A). No specific band was found when non-specific immunoglobulin (mouse IgG) was used for co-IP, and APP was confirmed in all co-IP samples (Fig. 4A). These results confirmed that sortilin has two binding sites to APP, one in ECD within 78–385 (N’-terminal part of the VPS10 domain) and the other in ICD. We further characterized the binding site of sortilin-ICD to APP and found that Sort-del.MS2 and Sort-MS1/MS2 were coimmunoprecipitated with 22c11, whereas Sort-del.MS1 and Sort-del.MS1/MS2 were not detected using the same antibody (Fig. 4B). In negative controls, neither the mouse IgG pulled down any specific band nor the anti-APP antibody immunoprecipitated pECFP from HEK293 cells co-expressing APP695 and pECFP (Fig. 4C). Thus, our data indicates that while the deletion of MS2 alone did not affect sortilin-ICD binding to APP, the deletion of MS1 however, completely abolished the interaction of sortilin-ICD with APP, suggesting that MS1 is a key binding site to APP.

In order to further validate the interaction between sortilin and APP, we performed the competitive co-IP assay using synthesized MS1 peptide or purified Sort-T-myc/His protein as the control competition in co-IP reaction. We monitored if the MS1 peptide and Sort-T effectively compete with the protein interaction. We found that Sort-T blocked Sort 78-385 binding to APP695, but not by BSA; and the MS1 peptide blocked Sort del.MS2 binding to APP695, but not by scramble MS1 peptide (Fig. 4D). Both BSA and scramble MS1 peptide were used as non-specific binding controls in the competitive co-IP. In addition, we made two new constructs, Sort-sp-78-385 and Sort-sp-del.MS2, which are similar to Sort-78-385CFP and Sort-del.MS2CFP but also includes a signal peptide. We used these constructs for the competitive co-IP and found the results were similar to when we used the constructs without a signal peptide, suggesting that these original constructs were not misfolded (Fig. 4E).

**Figure 5:**
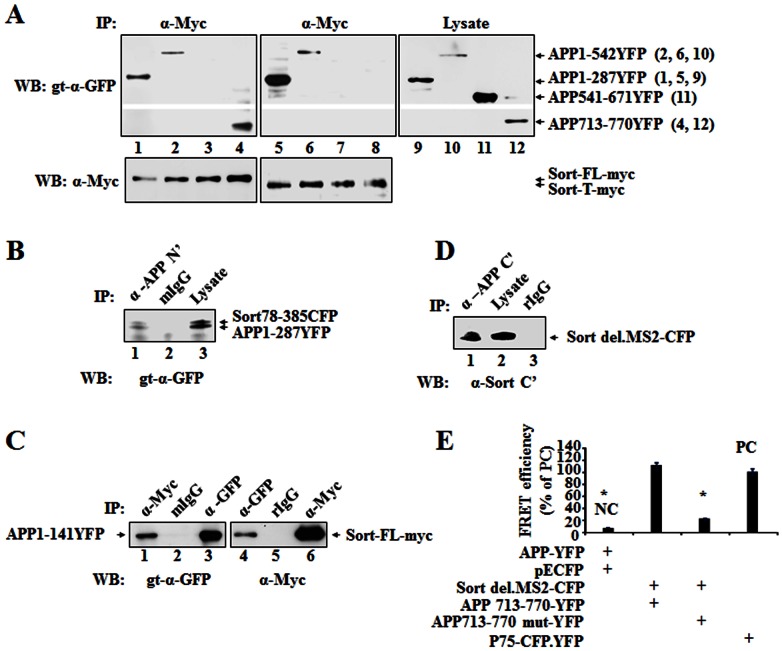
Mapping APP binding to sortilin and the binding sequences. (**A**) Determining APP 1-287 and APP 713-770 binding to sortilin. HEK293 cells were co-transfected with sort-FL-myc and APP 1-287YFP or APP 1-542YFP or APP 541-671YFP or APP713-770YFP (lanes 1–4). Sort-T-myc was used for the co-transfection with the same APP-YFP constructs (lanes 5–8). Lysates from each co-transfection were used as input (lane 9–12). Cell lysates immunoprecipitated with α-Myc (lanes 1–8) and inputs were blotted with goat (gt)-α-GFP. The co-IP blot was re-probed with α-Myc for sortilin constructs (lower). APP 1-542YFP (87 kDa), APP 1-287YFP (58 kDa), APP 541-671YFP (42 kDa), APP 713-770YFP (33 kDa), Sort-myc (115 kDa) and Sort-T-myc (110 kDa) are indicated by arrows. (**B**) Determining N terminal binding sites between APP and sortilin. HEK293 cells were co-transfected with APP 1-287YFP and Sort 78-385CFP. Cell lysates were immunoprecipitated with α-APP-N’ for APP1-287YFP (lane 1) and mouse IgG (mIgG) as a control for non-specific binding (lane 2), and blotted with gt-α-GFP. Lysate was used as input (lane 3). APP1-287YFP (58 kDa) and Sort78-385CFP (61 kda) are indicated by arrows. (**C**) Determining APP 1-141 binding to sortilin. HEK293 cells were co-transfected with APP 1-141YFP and Sort-FL-myc. Cell lysates were immunoprecipitated with α-Myc for Sort-FL-myc (lane 1) and mIgG (lane 2), and blotted with gt-α-GFP for APP1-141YFP. The direct Immunoprecipitated APP1-141YFP by α-GFP was used as input (lane 3). Also, cell lysates were immunoprecipitated with rabbit-α-GFP for APP1-141YFP (lane 4) and rabbite IgG (rIgG) as control (lane 5), and blotted with α-Myc for sortilin. The direct immunoprecipitated Sort-FL-myc by α-Myc was used as input (lane 6). APP1-141YFP (41 kDa) and Sort-FL-myc (115 kDa) are indicated by arrows. (**D**) Determining C terminal binding sites between APP and sortilin. HEK293 cells were co-transfected with APP 713-770YFP and Sort del.MS2-CFP. Cell lysates were immunoprecipitated with α-APP-C’ for APP 713-770YFP (lane 1) and rIgG (lane 3), and blotted with α-Sort C’. Lysate was used as input (lane 2). Sort del.MS2-CFP (34 kDa) is indicated by arrow. (**E**) Determining Sort-MS1 (del.MS2) interaction with APP NPTYKFFE motif. Sort del.MS2-CFP was used with APP 713-770-YFP or APP 713-770 mut-YFP for the co-transfection of HEK293 and then subjected to FRET. APP 713-770 mut-YFP construct contains a mutated NPTYKFFE motif where Y and F (underlined) are substituted with A. FRET efficiency representing the protein-protein interaction was determined from a photobleached region of interest (ROI). Three independent experiments were performed. Bars represent mean± SEM (n = 6 ROI×3). The star (*) indicates *p*<0.01. Abbreviation: negative control: NC; positive control: PC.

To determine which part of APP binds to sortilin, we performed co-IP in a similar way using each APP construct and Sort-FL. We found that APP 1-287, APP 1-542 and APP 713-770 were detected in the samples immunoprecipitated with anti-myc antibody but not APP 541-671 (Fig. 5A). However, only APP 1-287 and APP 1-542 were immunoprecipitated with Sort-T (Fig. 5A). The specificity of Co-IP was confirmed by using pEYFP, which showed Sort-FL did not interact with pEYFP (Fig. 1D). In addition, Sort-FL or Sort-T was detected in the co-IP samples (Fig. 5A). Taken together, the data demonstrate the intermolecular head-to-head and tail-to-tail interactions between sortilin and APP, and suggest that APP 1-287 and APP 713-770 interact with Sort 78-385 and Sortilin MS1, respectively. Indeed, we found that Sort 78-385 was immunoprecipitated with APP 1-287 by anti-APP N’ terminal antibody (Fig. 5B) and that Sort del.MS2 was immunoprecipitated with APP 713-770 by anti-APP C’ terminal antibody (Fig. 5D), whereas no specific band was shown when mouse and rabbit IgG as non-specific binding was used for co-IP. To further narrow down N’ terminal APP binding site to sortilin, two APP constructs, APP 1-141 and APP 141-287, were cloned and used for co-IP. It was determined that APP 1-141 was coimmunoprecipitated with sortilin-myc/His (sort-FL) by anti-myc antibody (Fig. 5C left panel) but not APP 141-287 (data not shown). Similar data resulted when using anti-GFP antibody for co-IP, which showed Sort-FL was coimmunoprecipitated with APP 1-141 (Fig. 5C right panel) but not with APP 141-278 (data not shown). Thus, our data indicate that APP 1-141 contained a binding site to sortilin 78-385.

In addition, our FRET data also showed that substitution of tyrosine (Y) and the last phenylalanine (F) with alanine (A) in NPTYKFFE motif (NPTY overlapping with YKFFE), of APP-ICD completely abolished the interaction between Sort-del.MS2 and APP 713-770, suggesting that the NPTYKFFE motif is important for APP-ICD to interact with sortilin MS1 (Fig. 5E). NPXpY within NPTYKFFE is an internalization signal for membrane proteins [26] and is involved in the interaction of several cytoplasmic proteins [27].

### Sortilin Regulates APP into Late Endosomes/Lysosomes

**Figure 6:**
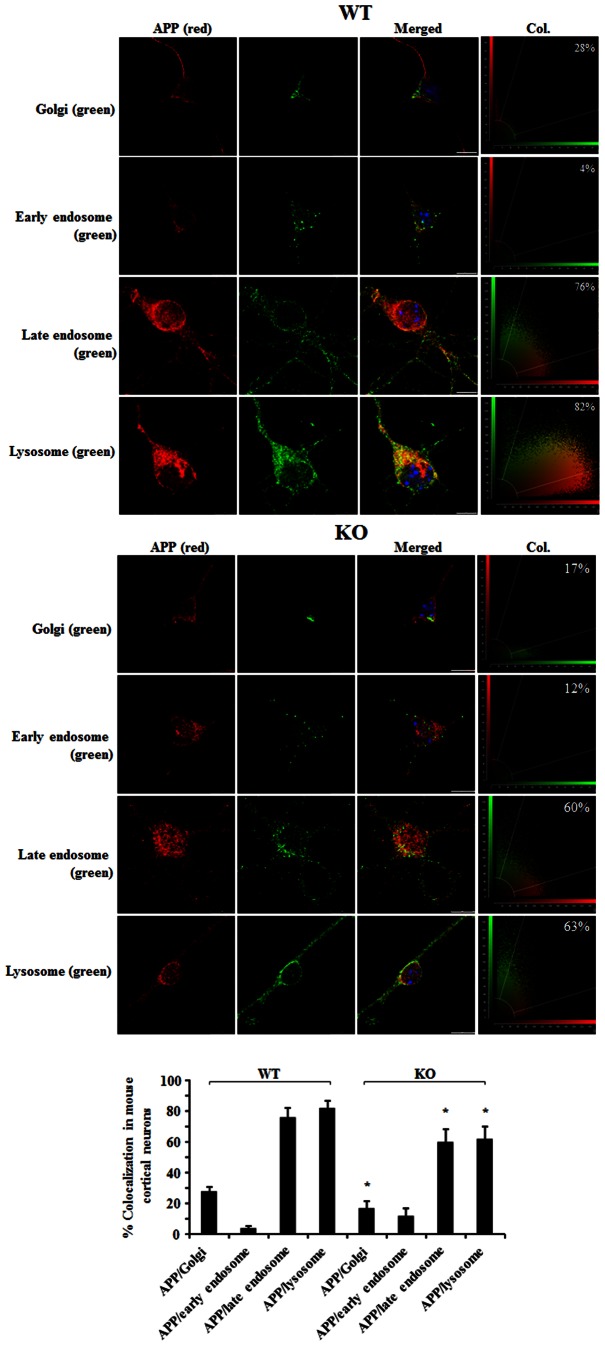
Lack of sortilin reduces APP distribution in lysosome and increases APP distribution in lipid rafts. Colocalization of APP with cell organelles in cortical neurons. Wild type (WT) and sortilin knockout (KO) mouse cortical neurons were immunostained for APP with mouse anti-APP-N’ (22c11) and followed by staining with Cy3 conjugated secondary antibodies (red) and cell organelles: Golgi, Early endosome, late endosome and lysosome immunostained with Giantin, EEA1, anti-mannose 6 phosphate receptor (for late endosome) and Lamp1, followed by staining with Alexa 488 conjugated secondary antibodies (green). Cell nuclei are stained by DAPI (blue). The colocalization is indicated in merged panels (yellow). Snapshots of colocalization are shown on the right of each panel. The percentage of colocalization (Col.) is plotted and is represented as mean± SEM (n = 20). The colocalization of APP with cell organelles is compared between WT and KO neurons. The star (*) indicates *p*<0.01. Scale bar 10 µm.

To analyse the APP intracellular distribution in more detail, we double-labelled WT and sortilin KO mouse cortical neurons with specific antibody to APP and to different cellular compartments (Fig. 6). We found that a small fraction of APP colocalized with Golgi complex in both WT (28%) and KO (17%) in cells. Also, a small amount of APP colocalized with early endosome marker (EEA1) in WT neurons (4%) and KO neurons (12%). Interestingly, a high proportion of APP was found to colocalize with late endosome marker (78%) in WT neurons, but the colocalization was significantly decreased to 60% in sortilin KO neurons (*p*<0.05). Moreover, 82% of APP was found to colocalize with lysosome marker in WT neurons, whereas the colocalization was significantly decreased to 63% in sortilin KO neurons (*p*<0.05). A significant decrease of colocalization of APP in late endosomes and lysosomes in sortilin KO neurons relative to WT neurons was further confirmed after the data was plotted quantitatively (Fig. 6). This data suggests that sortilin is involved in trafficking of APP in the endosome-lysosome pathway. Also, the colocalization between APP and Golgi complex was significantly higher in WT than KO. There was a noticeable change in terms of the colocalization of APP with EEA1 in both WT and KO neurons, but the detected change was too low to be informative for this study.

### Sortilin Regulates APP into Lipid Rafts

**Figure 7:**
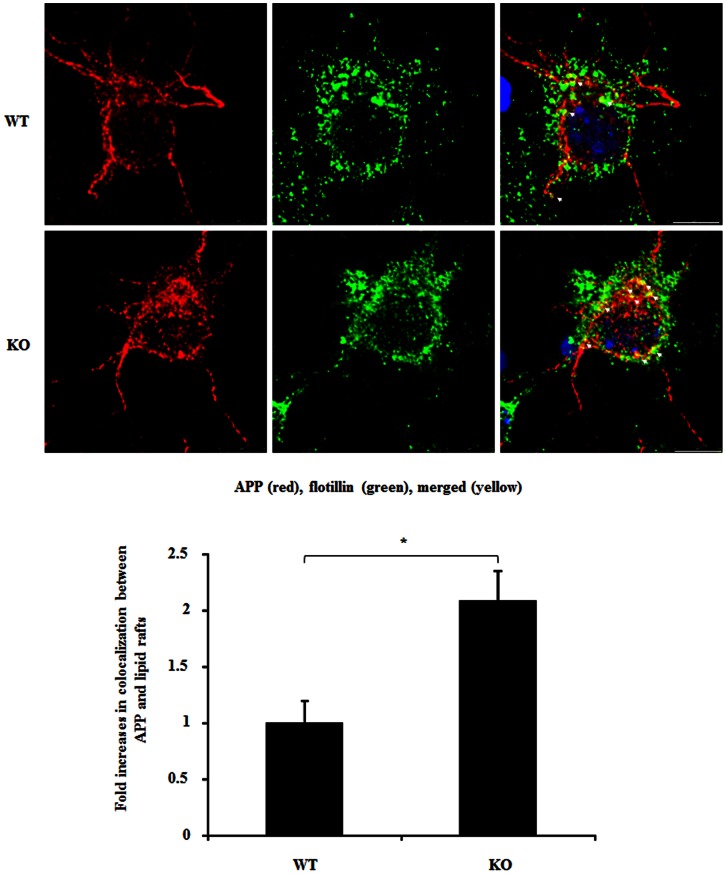
Lack of sortilin increases APP distribution in lipid rafts in cortical neurons. Wild type (WT) and sortilin knockout (KO) mouse cortical neurons were immunostained for APP with mouse anti-APP-N’ (22c11) and followed by staining with Cy3 conjugated secondary antibodies (red), and lipid rafts were immunostained with anti-flotillin, followed by staining with Alexa 488 conjugated secondary antibodies (green). Cell nuclei are stained by DAPI (blue). Colocalization is analyzed by counting the number of merged APP/raft lipids (yellow), and is plotted as mean of fold increase ± SEM (n = 20 neurons). The star (*) indicates *p*<0.01. Scale bar 7.5 µm.

To further determine if APP is possibly exposed to β- and γ-secretase in the presence or absence of sortilin, we investigated APP distribution in lipid rafts that involve high β and γ-secretase activities, Aβ production and aggregation [23], [28], [29]. We performed double-labelling of mouse cortical neurons with antibodies to APP and flotillin-1, a lipid raft component, and analyzed the colocalization of APP with lipid rafts by counting the number of merged spots in neurons. We found that sortilin KO neurons displayed a 2-fold increase in colocalization, over WT neurons (*p*<0.01), suggesting that APP is more likely to be associated with lipid rafts after deletion of sortilin (Fig. 7).

### Deletion of Sortilin MS1 Reduces APP Distribution in Lysosomes

**Figure 8:**
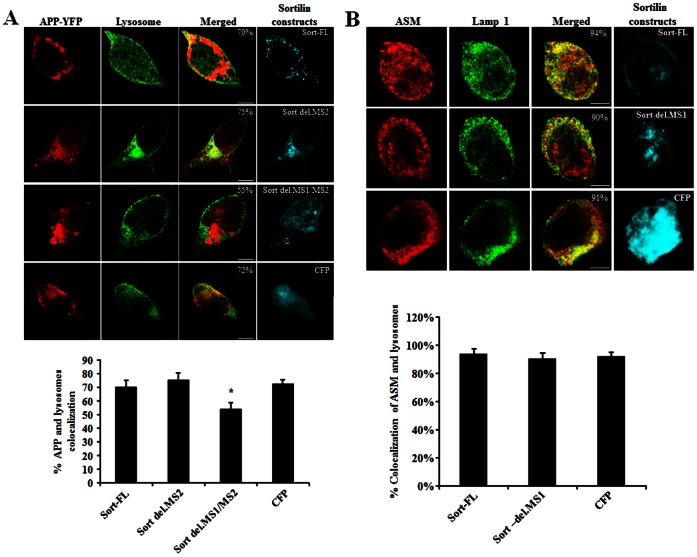
Effect of sortilin binding domain on APP lysosomal targeting. A: APP colocalization with lysosome. HEK293 cells were co-transfected with APP770-YFP and different sortilin constructs. The expressed APP and sortilin constructs excluding Sort-FL were visualized by either YFP or CFP fluorescence. Sort-FL-myc/His was immunostained with anti-sortilin, followed by staining with Cy3 conjugated secondary antibodies. Lysosomes were immunostained with lysosomal antibody (Lamp1), followed by staining with Cy5 conjugated secondary antibodies. B: Sortilin constructs sorting ASM to lysosomes. HEK293 cells were transfected with different sortilin constructs. The endogenous ASM was immunostained with mouse anti-ASM (Abcam), followed by staining with Cy3 conjugated secondary antibodies. Lysosome were immunostained with lysosomal antibody (Lamp1), followed by staining with Alexa 488 conjugated secondary antibodies. Sortilin constructs excluding Sort-FL were visualized by CFP fluorescence. Sort-FL-myc/His was immunostained with anti-sortilin, followed by staining with Cy5 conjugated secondary antibodies. Plotted colocalization is indicated at bottom. The percentage of colocalization is represented as mean± SEM (n = 20). The colocalization is compared with Sort-FL. The star (*) indicates *p*<0.01. Scale bar 7.5 µm.

As sortilin ICD showed a strong interaction with APP, we focused on characterising this binding site. To determine if the identified binding site of sortilin-ICD to APP influences APP lysosomal trafficking, we performed ICC using HEK293 cells co-expressing APP770-YFP and each varied sortilin-ICD construct (e.g., Sort del.MS2, Sort del.MS1/MS2 and Sort-FL). To avoid fluorescent dye (YFP and CFP) interfering, lysosomes was stained by Cy5. Our results showed Sort-FL and Sort del.MS2 yielded a cytoplasmic distribution of APP, whereas Sort del.MS1/MS2 caused APP aggregates in the preinuclear regions. Cells co-expressing pECFP showed a cytoplasmic distribution of APP similar to Sort-FL and Sort.del.MS2, suggesting that the expression of CFP (mock DNA) did not affect APP cellular distribution but Sort del.MS1/MS2 did. We also observed that high colocalization of APP with lysosome resulted from Sort-FL (70%), Sort del.MS2 (75%) and CFP (72%), whereas low colocalization of APP with lysosome was shown by Sort del.MS1/MS2 (55%) (Fig. 8A). The colocalization of APP with lysosome was significantly altered in the cells expressing Sort del.MS1/MS2, compared with Sort-FL or Sort del.MS2 (*p*<0.01). The question is if the formation of aggregates of APP in the cytoplasm, after transfecting the cells with Sort del.MS1/MS2 would or would not result in a general failure of the sorting process. To address this question, we examined the endogenous lysosomal trafficking of acid sphingomyelinase (ASM) [21] in HEK293 cells transfected with Sort-FL, Sort del.MS1 and pECFP, respectively. Our data indicated that Sort del.MS1 did not have a significant impact on the endogenous ASM trafficking to lysosomes, compared with Sort-FL (Fig. 8B), suggesting the sorting process was not altered in the transfected cells. Therefore, lack of MS1/MS2, especially the deletion of MS1 might impair APP lysosome trafficking and intracellular distribution.

### Deletion of MS1 or Sortilin Increases APP Distribution in Lipid Rafts

**Figure 9:**
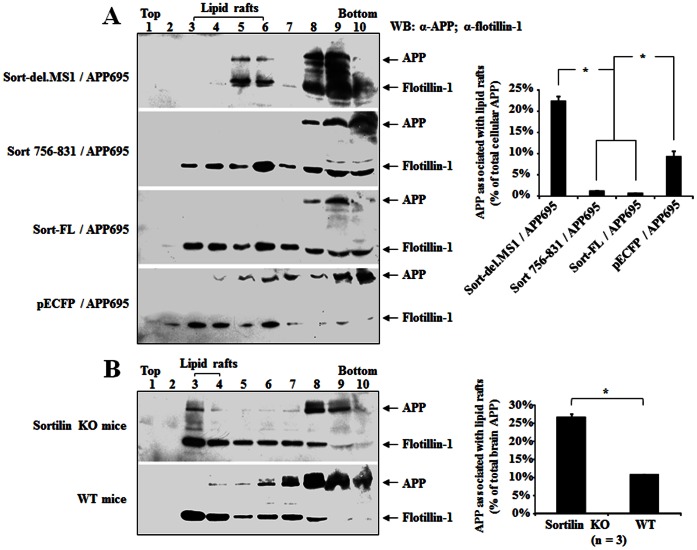
Effect of sortilin on APP distribution in lipid rafts. A: HEK293 cells co-transfected with APP695 and sortilin constructs were lysed in Triton-X-100 buffer and subjected to discontinuous sucrose density gradient ultracentrifugation fractionation. Equal volumes from each fraction were examined by WB for APP and flotillin-1. B: Lack of sortilin increases APP distribution within lipid rafts in sortilin KO mice. Mouse brains were homogenized in Triton-X-100 buffer and subjected to discontinuous sucrose density gradient ultracentrifugation fractionation. Equal volumes from each fraction were examined by WB for APP and flotillin-1. Bars represent mean± SEM (n = 3) from three independent experiments. The star (*) indicates *p*<0.01.

To further investigate if the binding site of sortilin-ICD to APP affects the distribution of APP in lipid rafts, we performed lipid raft fractionation using HEK293 cells co-transfected with APP695 and different sortilin constructs. We found that APP was detected in low density lipid raft fractions (3–6) in cells transfected with Sort-del.MS1 or pECFP (mock DNA), whereas APP was retained in high density fractions (8–10) in cells transfected with Sort 756-831 or Sort-FL (Fig. 9A). APP in lipid rafts was significantly increased in Sort-del.MS1 transfections, compared with Sort 756-831 and Sort-FL (*p*<0.01), suggesting that the deletion of sortilin MS1 can result in an increase of APP in lipid rafts, probably due to the redistribution of APP from high density fractions to lipid raft fractions. APP redistribution in mock DNA transfections may be due to overexpression of APP relative to sortilin. *In vivo* lipid raft fractionation studies also showed a significant increase of APP in lipid raft fraction (3–4) in the brain tissues of sortilin KO mice, compared with WT mice (*p*<0.01), indicating that a lack of sortilin causes APP redistribution to lipid rafts (Fig. 9B).

### Deletion of MS1/MS2 Reduces the Lysosomal Degradation of APP in Transfected HEK293 Cells

**Figure 10:**
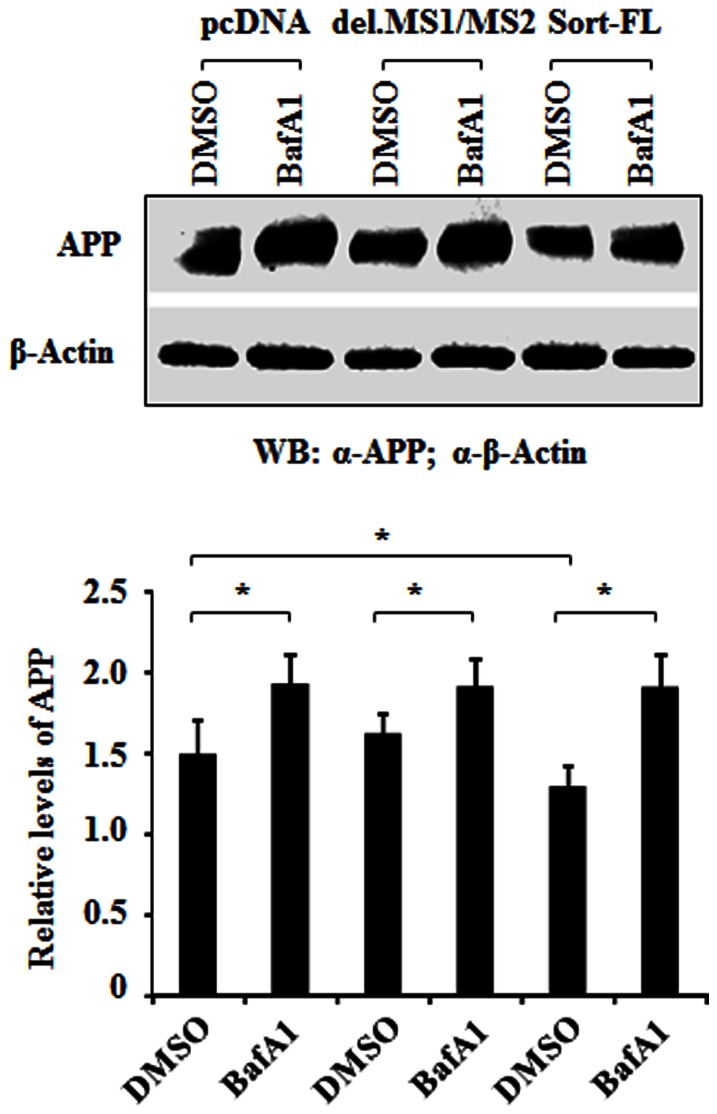
Effect of sortilin on APP lysosomal degradation. APP lysosomal targeting. HEK293 cells were co-transfected with APP-YFP and sortilin-pcDNA3.1 constructs or pcDNA3.1 (mock DNA) plasmid in 1∶1 molar ratio for 24 hours and then treated with DMSO or Bafilomycin AI, a lysosomal inhibitor, (BafA1, 4 µM, Sigma) for 6 h [30]. Cell lysates were harvested and APP level was examined by WB with mouse anti-APP-N’ (22c11). Transfected APP-YFP served to monitor transfection efficiency. The APP level was plotted after correction as to the corresponding β-actin and transfection efficiency.

To address if sortilin mediates APP to lysosomes for degradation, we examined if treatment with the lysosomal inhibitor bafilomycin A1 (BafA1) could rescue steady-state levels of APP in cells co-expressing either sort-FL or Sort del.MS1/MS2 [30]. Cells treated with BafA1 showed a significant increase of steady-state levels of APP in the absence of sortilin, consistent with previous reports that APP proteins are targeted for lysosomal degradation [6], [31], [32] (Fig. 10). Co-expression of Sort-FL yielded a significant decrease in steady state levels of APP, whereas co-expression of Sort-del. MS1/MS2 did not show any significant change of APP levels in untreated cells, compared with untreated controls (pcDNA). The expression of APP levels was significantly rescued by adding BafA1 to inhibit lysosomal activities (Fig. 10) (*p*<0.01). This data suggests that sortilin can transport APP to lysosomes for degradation and that MS1 and MS2 may play an important role in this process.

## Discussion

APP is well known for its cleaved products (Aβ, CTF and AICD), which are implicated in the pathogenesis of AD. Although the function of APP remains to be fully elucidated, understanding APP trafficking and processing would provide new insights into the regulatory mechanism of the amyloidogenic pathway. In this study, we investigated the role of sortilin in APP trafficking and distribution, and the underlying molecular mechanisms. We found that 1) sortilin is associated with APP via head-to-head (the extracellular domain) and tail-to-tail (the intracellular domain) interactions; and 2) sortilin regulates APP lysosomal and lipid raft trafficking through FLVHRY motif.

### The Interaction between Sortilin and APP is Important for APP Intracellular Distribution Including Lysosomal Targeting and the Distribution in Lipid Rafts

Our results show that sortilin is involved in APP intracellular distribution via the interaction between sortilin and APP. Our mapping study has defined that sortilin interacts with APP through each ICD and ECD. The sortilin N-terminal binding site in ECD to APP is identified within amino acid 78-385, which contains part of the Vps10p domain. However, the Vps10p domain of SorLA has shown no interaction with APP [33], suggesting this binding site is unique to sortilin. Since SorLA has been reported to interact with APP via the luminal domain [34] and the cytoplasmic tail [35], it is suggested that sortilin is likely to have a second binding site to APP. Indeed, we have identified that the sortilin C-terminal binding site to APP is FLVHRY (MS1) belonging to the F/YXXXXF/Y motif because the deletion of the FLVHRY abolishes the interaction between sortilin-ICD and APP-ICD. Given the fact that the F/YXXXXF/Y motif exists in SorLA and acts as an internalization/sorting signal in mannose-6 phosphate and low-density lipoprotein receptors [15], this interaction between APP and the FLVHRY motif highlights the potentially important role of sortilin in APP trafficking and processing. This is further supported by our *in vivo and in vitro* data which shows an altered distribution of APP in lysosomes and lipid rafts in neurons of sortilin KO mice, and in HEK293 cells co-expressing the sortilin MS1 deletion constructs. The APP-NPXYKFFE motif is likely to be responsible for the C’-terminal interaction with sortilin FLVHRY, because mutation of the NPXYKFFE motif eliminates this interaction. Recent reports have shown that mutant YKFFE motif (overlap with NPXYKFFE) is associated with redistribution of intracellular APP [36], [37], which could be a result of the disruption of the interaction between sortilin FLVHRY and APP NPXYKFFE. It is possible that the sortilin-ICD is involved in controlling the fate of intracellular APP. Phosphorylation of APP at T668, located 14 amino acids toward the N-terminal end from the ^681^GYENPTY^687^ motif, has been reported to regulate the interaction of the ^681^GYENPTY^687^ motif with other binders [38], suggesting that the phosphorylation may be involved in the interaction between sortilin FLVHRY and APP NPXYKFFE. The NPXYKFFE, containing a core NPXpY element, mediates APP internalization as well as the interaction between the proteins bearing phosphotyrosine-binding domains (such as Fe65, Shc, X11, mDab1 and JIP) [36], [39]. There is evidence to suggest that JIP is associated with the kinesin-1 motor [40], [41]. Sortilin also interacts with HAP1, a protein interacting with the p150glued subunit of dynactin [41], which regulates neurotrophin axonal trafficking [22], [42]. The interactions between these proteins can facilitate appropriate APP axonal transport and is important to sort APP into the non-amyloidgenic pathway [27]. Further studies are warranted to determine if the phosphorylation of APP at T668 plays a crucial role in the interaction with sortilin FLVHRY and if the N-terminal binding site of sortilin to APP has any roles in APP trafficking and processing. Nonetheless, our data indicates that the head-to-head and tail-to-tail interactions are required for sortilin/APP cellular events.

### Sortilin and its ICD may Play a Role in APP Processing by Regulating APP Trafficking to Lysosome and Lipid Rafts

The sortilin-ICD, especially the FLVHRY motif, plays an essential role in APP intracellular trafficking to the appropriate organelles. We found that a lack of sortilin and co-expression of sortilin constructs do not affect the distribution of APP in ER (data not shown), thus sortilin is not required in APP synthesis and APP/ER exit. However, the loss of sortilin or the deletion of FLVHRY causes a significant increase of APP in lipid rafts, but a decrease of APP in lysosome of neurons (Fig. 6, 7, 8A and 9), suggesting that sortilin can regulate APP into lysosomes and reduce the β and γ-secretase cleavage of APP in lipid rafts. In fact, the effect of sortilin on protein sorting to lysosomes has been described previously [6], [43]. The lysosomal trafficking of the sphingolipid activator proteins, β-hexosaminidase and β-glucuronidase is mediated by sortilin through Golgi-localizing, gadaptin ear homology domain, ARF-binding proteins adaptor protein (GGA). The cytoplasmic tail of sortilin also contains a dileucine motif that binds the GGA [12]. Therefore, it is possible that sortilin can mediate APP lysosomal targeting in a similar manner. We further demonstrated that sortilin mediates APP to lysosomes for degradation, where the FLVHRY (MS1) motif appears to be essential (Fig. 10A). This motif is responsible for the interaction with APP, but the dileucine motif may provide molecular bridging and flanking sequences for context in regulating APP trafficking and processing in cells. In addition, co-expression of Sort-del.MS1/MS2 is associated with the formation of APP aggregates in the cytoplasm in the transfected cells, raising the possibility that this would cause a general failure of the sorting process. By testing another protein, ASM known to be sorted to lysosomes by sortilin, we have demonstrated that the formation of APP aggregates is not the consequence of the general failure of the sorting process (Fig. 8B). Similar results have also been reported in TGF-b lysosomal targeting mediated by sortilin mutant constructs [30].

It is generally believed that SorLA retains APP in TGN, which has contributed to the non-amyloidogenic pathway [34]. However, sortilin may also function to prevent APP into the amyloidogenic pathway. A lack of the sortilin function would lead to an abnormal routing of APP trafficking. For example, an increase of APP distribution was found in lipid rafts in sortilin KO neurons as oppsed to WT neurons, suggesting the involvement of amyloidogenic processing of APP [44], [45]. Also, high activity of β-secretase and Aβ production have been reported in TGN, cellular organelles and lipid rafts [8], [23], [28], [46], suggesting sortilin negatively regulates Aβ production. Indeed, our results showed that the deletion of the FLVHRY motif and lack of sortilin caused an increase of APP in lipid rafts and a decrease of APP in lysosomes (Fig. 6, 7 and 9), suggesting that sortilin and this motif are more likely to affect APP processing. We are aware that an increase in Aβ was previously reported in a sortilin stable transfected cell-line [20]. The explanation of the discrepancy might be accounted for by a variety of factors, including difference in the experimental design, cell types and analysis. However, the results from the deletion of MS1 are in agreement with that reported by the same group, showing a significant increase in Aβ by deleting sortilin ICD. Our data from the present study has implied that the effect of sortilin on APP intracellular distribution directly contributes to maintaining the balance of Aβ production, as a false routing and/or a prolonged exit from Golgi-TGN and/or the cellular membrane have been related to altered APP processing, with increased Aβ production. Thus, the interaction between sortilin and APP through the FLVHRY motif is vital for APP trafficking and processing. However, more studies are needed to determine if sortilin and this motif are directly involved in the amyloidogenic pathway.

In conclusion, we are the first to demonstrate head-to-head and tail-to-tail interactions between sortilin and APP. The FLVHRY motif-mediated interaction is crucial for APP lysosomal and lipid raft distribution, and may promote lysosome-dependent degradation of APP. Our data provides new molecular insights into APP trafficking and processing.
